# Tomographic findings and mortality in patients with severe and critical pneumonia with COVID-19 diagnosis

**DOI:** 10.1016/j.rmcr.2022.101752

**Published:** 2022-10-05

**Authors:** O. Jiménez-Zarazúa, L.N. Vélez-Ramírez, A. Hernández-Ramírez, B.I. Arévalo-Rivas, M.A. Galván-Casas, G.U. García- Zavala, J.D. Mondragón

**Affiliations:** a Hospital General de Zona IMSS No. 21, Department of Internal Medicine, León, Guanajuato, Mexico; b Universidad de Guanajuato, Department of Medicine and Nutrition, León, Guanajuato, Mexico; c Hospital General León, Department of Radiology, León, Guanajuato, Mexico; d Universidad de Guanajuato, Department of Medicine and Nutrition, Graduate School Coordiinator, León, Guanajuato, Mexico; e Hospital General de Zona IMSS No.58, Hospital Director, León, Guanajuato, Mexico; f University of Groningen, University Medical Center Groningen, Department of Neurology, Groningen, the Netherlands; g University of Groningen, University Medical Center Groningen, Alzheimer Center Groningen, Groningen, the Netherlands

**Keywords:** Acute respiratory distress syndrome, COVID-19, Mechanical ventilation, Mortality, Pulmonary computed tomography

## Abstract

**Introduction:**

A high percentage of patients with non-severe (17.9%) and severe (2.9%) atypical pneumonia do not display pulmonary tomographic findings upon hospital admission; furthermore, lesion associated with COVI-19 are peripherally distributed in a multifocal ground-glass pattern, as well as displaying an irregular consolidation pattern, with a posterior or lower lobe predilection. The main objective of this study was to identify the pulmonary radiological patterns in patients diagnosed with SARS-CoV-2 pneumonia, the factors associated with the need for mechanical ventilation, as well as their survival rates at 30 days.

**Methods:**

We report the pulmonary tomographic findings of 490 consecutive patients with severe and critical pneumonia due to SARS-CoV-2. The patients were classified according to the tomography and demographic findings, sepsis severity prognostic scales, Charlson comorbidity index (CCI), the Sequential Organ Failure Assessment (SOFA), and the Acute Physiology and Chronic Health Evaluation (APACHE IV). The Kaplan-Meier method was used to calculate survival distributions.

**Results:**

89.80% of patients had ground-glass opacities, 81.63% radiologic consolidation sign, 42.45% vascular thickening pattern, 37.55% lymphadenopathies, 14.90% pleural effusion, and 2.65% pulmonary thrombosis; meanwhile, 91.02% had bilateral lesions, 85.51% had peripheral lesions, and 75.92% had basal lobe lesions. APACHE IV (HR, 1.191, 95% CI [1.126, 1.260]), SOFA (HR, 5.178, 95%CI [3.103, 8.641]), and CCI (HR, 0.673, 95%CI [0.510, 0.889]), as well as the pulmonary damage severity index (HR, 1.282, 95%CI [1.151, 1.428]), predict the need for invasive mechanical ventilation. Only moderate ARDS patients with mild and severe lung disease showed different 30-day mortality distributions (χ^2^ = 7.00, p = 0.008).

**Discussion:**

Although the survival distributions did not vary significantly, an overwhelming majority of patients (i.e., 84.35%) with a higher pulmonary damage severity index (i.e., 23>) died within 30 days of hospital admission, while only 25.91% with moderate lung damage and 2.42% with mild lung damage.

## Highlights

• Mortality at 30 days was 84.35% for patients with a high pulmonary damage.
• Mortality at 30 days was 25.91% for patients with moderate lung damage.
• Mortality at 30 days was 2.42% for patients with mild lung damage.
• Most patients with COVID-19 had ground-glass opacities and radiologic consolidation sign.

## 1 Background

At the end of 2019, a new coronavirus was linked to several cases of pneumonia in the city of Wuhan in the Hubei province of China [1]. The virus that causes COVID-19 is called severe acute respiratory syndrome coronavirus 2 (SARS-CoV-2); previously, it was called 2019-nCoV [1,2]. Like MERS-CoV disease, there is still no specific treatment for SARS-CoV-1 and -2 disease [3]. The recommended management for SARS-CoV-2 infection is isolation and supportive care, including oxygen therapy, invasive mechanical ventilation, fluid management, steroid administration, and antibiotic treatment for secondary bacterial infections [[4], [5], [6], [7]].

The use of computed tomography should be considered as the first option for imaging diagnosis in patients with suspected pneumonia [8]; highlighting the importance of recognizing the tomographic radiological patterns associated with patients with pneumonia. Pulmonary tomographic findings in patients with SARS-CoV-2 pneumonia have reported that a peripherally distributed multifocal ground-glass pattern may occur with an irregular consolidation pattern, with a posterior or lower lobe predilection [[9], [10], [11]]. However, imaging diagnosis is difficult for patients with atypical pneumonia since 17.9% of patients with non-severe disease COVID 19 do not display pulmonary tomographic findings upon hospital admission, while 2.9% in patients with severe disease [5]. We report a series of 490 consecutive cases in which we report the pulmonary tomographic findings of patients with severe and critical pneumonia due to SARS-CoV-2. The patients were classified according to the tomography and demographic findings, prognostic scales, and biomarkers. First, pulmonary radiological patterns were identified in patients diagnosed with SARS-CoV-2 pneumonia, followed by the assessment of the association between disease severity scales and invasive mechanical ventilation. A secondary objective was to identify the mortality at 30 days associated with the type of pneumonia (i.e., severe or critical), acute distress syndrome (i.e., ARDS levels), and different levels of lung disease (i.e., pulmonary damage severity index).

## 2 Methods

An observational and retrospective study was conducted that included a consecutive case series of patients with SARS-CoV-2 (i.e., Polymerase chain reaction, PCR, confirmed diagnosis) pneumonia at the internal medicine department of our hospital (Hospital General Regional N. 58 IMSS and Hospital General de Zona N. 21 de León, León, Mexico) from June 2020 to March 2021. The inclusion criteria included: 1) patients older than 18 years; 2) both sexes; 3) patients with contrasted chest computed tomography at the time of taking the sample to make the confirmatory diagnosis by PCR (i.e., ≤24 hours); 4) patients with a PCR positive test for SARS-CoV-2 (i.e., confirmed by the National Network of Public Health Laboratories, Institute for Diagnosis and Epidemiological Reference, Mexico City); and 5) patients with a complete file with progress notes up to 30 days of hospitalization. The exclusion criteria were: 1) patients with a negative PCR test for SARS-CoV-2; 2) patients with a diagnosis of pulmonary neoplasia or evidence of a pulmonary metastatic process; 3) patients with autoimmune disease (e.g., systemic lupus erythematosus, rheumatoid arthritis, polymyositis, dermatomyositis, Sjögren's syndrome); 4) patients with a previous medical diagnosis of interstitial disease (e.g., idiopathic pulmonary fibrosis, mixed connective tissue disease, collagen disease, sarcoidosis, respiratory bronchiolitis associated with interstitial disease, unspecified interstitial pneumonia, unclassifiable interstitial disease) [12]; 5) pregnant patients; and 6) patient who refused to undergo pulmonary CT scan. Meanwhile, the elimination criteria were: 1) patients without a complete medical file necessary to extract the variables of interest for this study; and 2) patients lacking a 30-day follow-up. Upon hospital admission, the patient signed an informed consent permitting the use of her clinical file information for didactic, research, and publication purposes. This study was approved by the Institutional Review Board (IRB) of our hospital on June 15th, 2020. The IRB approval number: F-2020-1008-038 and ClinicalTrials.gov identifier: NCT04499378. Abiding by the Declaration of Helsinki, patient anonymity was guaranteed.

The images were processed with a Toshiba 16 multislice tomograph and by a Siemens 16 multislice tomograph, which is scaled to 32 multislice. The pixel spacing of CT images was 0.72 mm and 0.85 mm for the uCT and Siemens scanner (Erlangen, Germany), respectively. The slice thickness was 5 mm for both scanners, all scans were evaluated by two radiologists. A nasopharyngeal sample was collected for each patient at the time of admission to the emergency department with a RT-qPCR SARS-COV2 kit Superscript III platinum one-step quantitative RT-PCR system (Invitrogen, Thermo Fisher Scientific, Waltham, MA USA).

Demographical variables such as sex, age, weight, height, body mass index, as well as clinical variables such as length of stay in the hospital, intra-hospital stay, vital signs, comorbidities, heart failure, acute kidney failure, acute respiratory failure, laboratory work-up (e.g., serum lactate, PaO_2_/FiO_2_, arterial gases, D-dimer, lactate dehydrogenase, platelets, leukocytes, liver function enzymes, creatinine, albumin, hemoglobin, C-reactive protein), and therapeutic management (e.g., use of vasopressors and inotropes, renal replacement therapy, steroid use, mechanical ventilation, antibiotic treatment) were extracted from the medical file. Sepsis severity was assessed with the chronic health status through the Charlson comorbidity index (CCI), the Sequential Organ Failure Assessment (SOFA), and the Acute Physiology and Chronic Health Evaluation (APACHE IV).

**Fig. 1:**
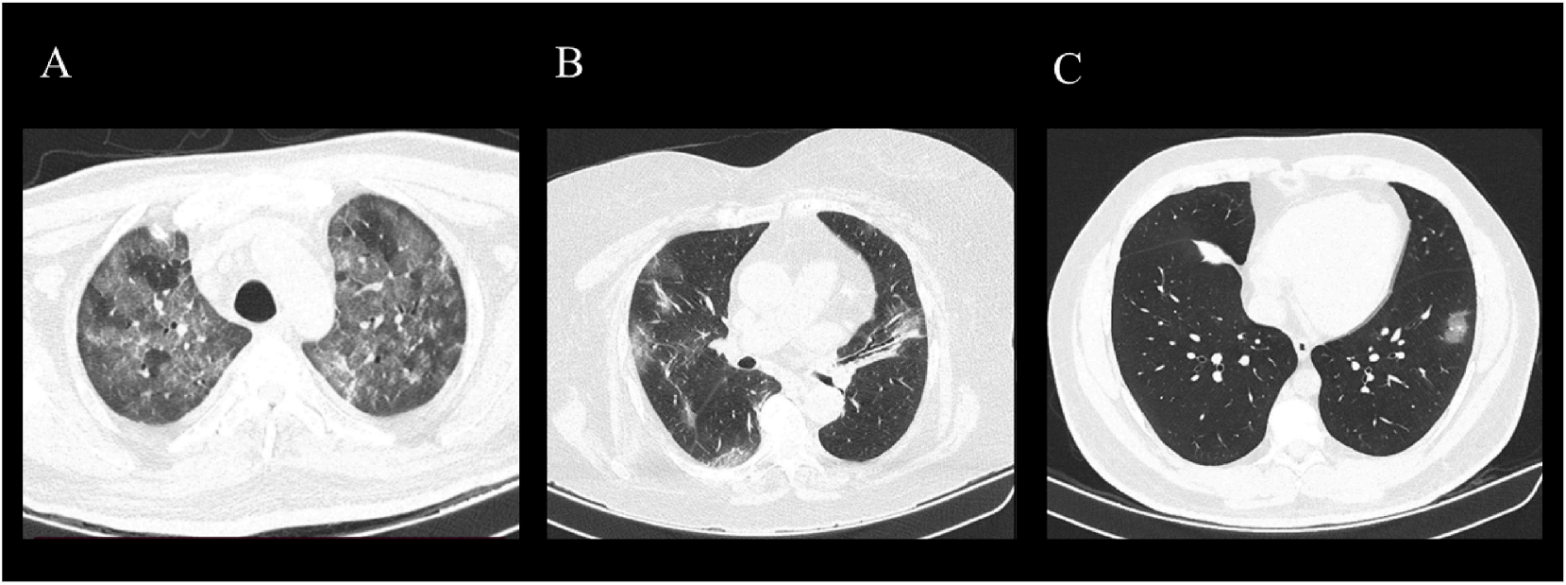
Tomographic findings A) Computed tomography, axial cut of the lung. Severe affection of the lung, both superior lung lobules are seen with a diffuse ground-glass image with air entrapment. Interlobular septal thickening, as well as vascular thickening pattern. B) Computed tomography, axial cut of the lung. Moderate affection of the lung, ground-glass zones with subpleural distribution are observed, with interlobular septal thickening and vascular thickening. C) Computed tomography, axial cut of the lung. Mild affection of the lung, small ground-glass opacities in the lower left lobule are observed.

The following criteria were used to classify pneumonia as severe: 1) respiratory distress, ventilatory rate >30 breaths/min; 2) at rest, finger-clip oxygen saturation <93%; and 3) PaO_2_/FIO_2_<300 mmHg. While critical pneumonia was defined by the following criteria: 1) respiratory failure requiring mechanical ventilation; 2) shock; and 3) other organ failures requiring Intensive Care Unit (ICU) monitoring [11]. Imaging classification was performed by two independent radiologists (Cohen's kappa = 0.89), and diagnostic and classification discrepancies were resolved via a third party and a consensus between the three radiologists. The blinded radiological classification initially dichotomized the CT scans as suggestive of COVID-19 or not suggestive of COVID-19. The tomographic findings were classified as 1) consolidation pattern, 2) ground-glass pattern, 3) nodular pattern, 4) presence of ganglia, 5) presence of thrombosis, and 6) vascular thickening pattern (Fig. 1). Furthermore, pattern and lesion location were recorded (i.e., right, left, or bilateral lung involvement, peripheral distribution, or basal distribution). A pulmonary damage severity index was calculated based on the estimated percentage of lung involvement based on the following criteria: a) score 0, 0% involvement; b) score 1, less than5% involvement; c) score 2, 5%–25% involvement; d) score 3, 26%–49% involvement; e) score 4, 50%–75% involvement; and f) score 5, greater than 75% involvement [13,14]. Each lobe was scored using these criteria, thus a total possible score of 0–25 was obtained for each patient at the time of ICU admission (i.e., <24hrs after positive PCR test for SARS-CoV-2).

Statistical analysis was performed using SPSS 25 (IBM Corp, Armonk, NY). Data were screened for outliers and normality assumptions. The normality of continuous variables (i.e., age, BMI, length of stay in the hospital, CCI, SOFA, APACHE IV) was assessed with the Shapiro-Wilk normality test and visually using histograms and QQ plots. Demographical and clinical factors are summarized using proportions and percentages. The variable sex, a categorical demographical variable, and the time to death, a clinical continuous variable, were assessed for statistical inference with a Mann-Whitney test. The clinical continuous variables (i.e., age, BMI, length of stay in the hospital, CCI, SOFA, APACHE IV, and pulmonary damage severity index) were assessed for statistical inference individually using independent-sample ANOVAs. Statistical significance was set at p < 0.0056 after a Bonferroni correction for multiple comparisons and effect sizes for the ANOVAs are reported as Eta squared (η^2^). A multiple logistic regression model with backward stepwise elimination was performed for the need for mechanical ventilation as the outcome measure. An interaction term between APACHE IV and SOFA was added for the clinical model. The Omnibus Test of Model Coefficients was used to calculate overall model fitness and change between models. The −2 log-likelihood statistic (–2LL) was used to assess if the predictor contributed to the overall model. R_L_^2^ or Hosmer-Lemeshow R^2^ was computed using (–2LL_baseline_) – (–2LL_new_)/–2LL_baseline_; the Cox-Snell R^2^ and Nagelkerke R^2^ are also reported as effect sizes for the logistic regression model. Statistical significance was set at p ≤ 0.05. The Kaplan-Meier method was used to calculate survival distributions; meanwhile, the Gehan-Breslow-Wilcoxon method was used to compare the equality of survival distributions, as it gives more weight to deaths at early time points. Both an overall (i.e., differences between all groups) and a between-group (i.e., differences found between-groups accounting for all between-group comparisons) survival analysis was performed. Overall, tests for the equality of survival times, as well as pairwise comparisons (i.e., between-group comparisons) were performed with statistical significance set at p < 0.05.

## 3 Results

Four hundred and ninety consecutive patients (286 males) were recruited for this study, with a mean age of 60.46 (±14.63, 16–97) and a mean body mass index of 28.5 (±2.99, 19.6–40.4). Clinically, 324 patients were classified as having severe pneumonia, while 166 had critical pneumonia. Furthermore, 251 patients did not have acute respiratory distress syndrome (ARDS), while 118 had mild, 86 had moderate, and 35 had severe ARDS. The type of lesion observed was: 440 patients had a ground glass image; 400 patients had radiological signs of consolidation; 73 patients had pleural effusion; lymphatic ganglia could be observed in 184 patients; 208 patients had a vascular thickening pattern; 13 patients exhibited evidence of thrombosis. Lesions were observed predominately bilaterally (i.e., 446), while 44 patients had unilateral lesions (i.e., 27 right). Meanwhile, in 419 patients the lesions were primarily observed in the periphery and in 372 patients the tomographic distribution of the lesions was observed in the basal lobules. Furthermore, the median pulmonary damage severity index score was 19 (mode, 25; interquartile range, 15–23), with 93 patients having a maximum of 25 (i.e., 19% of the sample). Lung damage was categorized into three groups based on the pulmonary damage severity index score (i.e., mild, 1–15; moderate, 16–22; and severe, 23–25). One hundred and twenty-four patients had mild lung damage, 220 had moderate, and 146 had severe pulmonary damage observed in their pulmonary CT scan.

## 4 Invasive ventilation

One hundred and fifty-four patients required invasive ventilation. First, between-group differences were assessed between patients who required mechanical ventilation and those who did not. Four prognostic scales were evaluated to assess the need for invasive mechanical ventilation (i.e., APACHE IV, SOFA, CCI, and pulmonary damage severity index). The group means of all four severity scales were different between patients who required ventilation and those who did not (APACHE, η^2^ = 0.575, p ≤ 0.001; SOFA, η^2^ = 0.606, p ≤ 0.001; CCI, η^2^ = 0.017, p = 0.004; pulmonary damage severity index, η^2^ = 0.299, p ≤ 0.001). After assessing between-group differences, a logistic regression was performed to assess the predictive value of each disease severity scale. Table 1 summarizes model coefficients and effect sizes of each scale in this population. Briefly, a higher SOFA score had the greatest impact on the need for invasive mechanical ventilation (χ^2^ = 39.61, p ≤ 0.001, HR 5.178, 95CI [3.103, 8.641]); furthermore, as the pulmonary damage severity index increased (χ^2^ = 20.49, p ≤ 0.001, HR 1.282, 95CI [1.151, 1.428]) and APACHE IV increased (χ^2^ = 37.36, p ≤ 0.001, HR 1.191, 95CI [1.126, 1.260]), the risk of mechanical ventilation did as well. Conversely, the fewer comorbidities (i.e., lower CCI) the lower the risk of needing mechanical ventilation (χ^2^ = 7.80, p = 0.005, HR 0.673, 95CI [0.510, 0.889]). After evaluating the risk factors associated with the need for invasive mechanical ventilation a survival analysis was performed. The overall survival between patients who required mechanical ventilation and those who did not were compared, with no statistically significant differences observed (χ^2^ = 0.026, p = 0.873, Supplementary Fig. 1).

**Table 1:** Logistic regression model coefficients and effect sizes.

Mechanical ventilation (n = 154)									
				95% CI for Odds Ratio			Pseudo-R^2^		
Variable	χ^2^	p	b	Lower	Odds	Upper	H&L	C&S	Negelkerke
APACHE IV	37.36	≤0.001	0.175	1.126	1.191	1.260	.130	.486	.683
SOFA	39.61	≤0.001	1.644	3.103	5.178	8.641	.257	.528	.741
APACHE IV * SOFA	29.82	≤0.001	−0.24	0.967	0.976	0.985	.353	.557	.782
Charlson comorbidity index	7.80	0.005	−0.395	0.510	0.673	0.889	.377	.564	.792
Pulmonary damage severity index	20.49	≤0.001	0.249	1.151	1.282	1.428	.455	.586	.823

CI: confidence interval. χ^2^: Wald test. Beta value refers to the measure of the modeled effect that reflects the parameter estimate. All reported p-values are corrected with a Bonferroni correction for multiple comparisons. H&L: Hosmer & Lemeshow R^2^. C&S: Cox & Snell R^2^. APACHE IV: Acute Physiology and Chronic Health Evaluation. SOFA: Sequential Organ Failure Assessment. CT: computed tomography.

## 5 Severe versus critical pneumonia

The group means of all four severity scales were different between patients who had severe and critical pneumonia upon admission to the critical care department (APACHE, η^2^ = 0.348, p ≤ 0.001; SOFA, η^2^ = 0.417, p ≤ 0.001; CCI, η^2^ = 0.033, p = 0.004; pulmonary damage severity index, η^2^ = 0.149, p ≤ 0.001). After assessing between-differences among the type of pneumonia a mortality analysis was performed, with no statistical differences were observed in the survival distributions of patients with severe and critical pneumonia (χ^2^ = 0.317, p = 0.573, Supplementary Fig. 2). To explore the role of lung damage as a predictor of survival at 30 days the pulmonary damage severity index was used. First, the survival distributions between patients with severe and critical pneumonia were performed. Out of the 324 patients with severe pneumonia, 107 had mild lung damage with one death after 30 days, 163 had mild lung damage with 15 deaths, and 54 had severe lung damage with 33 deaths. Meanwhile, patients out of the 166 patients with critical pneumonia, 17 had mild lung damage and 2 deaths, 57 had moderate lung damage and 42 deaths, and 92 had severe lung damage with 90 of those patients dying with 30 days. Although mortality increased with clinical severity and lung damage, the survival distributions were not statistically different (i.e., severe: χ^2^ = 1.889, p = 0.389; critical: χ^2^ = 0.696, p = 0.706, Supplementary Fig. 3).

## 6 Acute respiratory distress syndrome

The group means of all four severity scales were different between patients with different levels of acute respiratory distress upon admission to the critical care unit (APACHE, η^2^ = 0.303, p ≤ 0.001; SOFA, η^2^ = 0.372, p ≤ 0.001; CCI, η^2^ = 0.020, p = 0.018; pulmonary damage severity index, η^2^ = 0.166, p ≤ 0.001). Furthermore, the overall survival analysis at 30 days as a function of the degree of acute respiratory distress syndrome was assessed. No statistically significant differences were observed in the overall survival distributions between the four ARDS classifications (χ^2^ = 2.441, p = 0.446, Supplementary Fig. 4). Further analysis was performed to assess if different ARDS levels had different mortality distributions. An analysis of the survival distributions was performed comparing lung damage in patients with different levels of ARDS. While 251 patients had no ARDS, of those 96 had mild lung damage and one died, 113 had moderate lung damage and 10 died, while 42 had severe lung damage with 24 deaths among those patients. Among patients with mild ARDS (n = 118), 22 had mild lung damage and one died, 59 had moderate lung damage and 13 died, while 37 had severe lung damage with 32 patients dying. Patients with moderate ARDS (n = 86) had the following lung severity distribution and the number of deaths: 6 with mild lung damage and one death, 34 with moderate lung damage and 21 deaths, and 86 with severe lung damage and 68 deaths. Meanwhile, patients with severe ARDS had the following lung severity distribution: 14 with moderate damage and 13 deaths, 35 severe damage and 34 deaths. Similarly, to the survival distribution associated with pneumonia type, the mortality among the different ARDS groups was not significantly different (i.e., no ARDS: χ^2^ = 2.145, p = 0.342; mild ARDS: χ^2^ = 1.718, p = 0.424; moderate ARDS: χ^2^ = 4.719, p = 0.094; severe ARDS: χ^2^ = 3.375, p = 0.066).

## 7 Survival as a function of lung damage

**Fig. 2:**
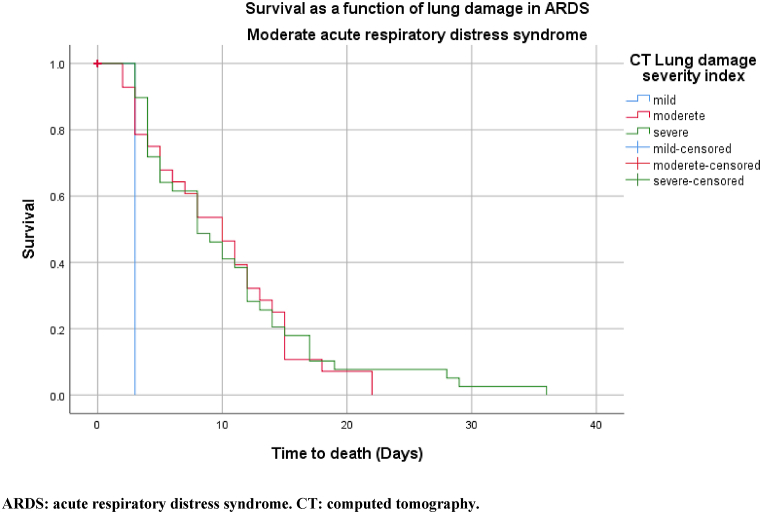
Survival as a function of lung damage in acute respiratory distress syndrome ARDS: acute respiratory distress syndrome. CT: computed tomography.

Finally, when the survival distributions among the different levels of lung damage are considered, no differences in survival distributions are observed (χ^2^ = 1.064, p = 0.588). Out of 124 patients with mild lung damage, 3 died within 30 days, while 57 patients out of the 220 with mild lung damage died and 123 out of 146 patients with severe lung damage perished within 30 days. The association between ARDS, the pulmonary damage severity index, and survival at 30 days, was also assessed and is reported in Table 2. The comparison between survival distributions for each ARDS classification and pulmonary damage severity index is presented in Table 3. In moderate ARDS patients, the survival distributions between mild and severe lung damage were statistically different (χ^2^ = 7.00, p = 0.008, Fig. 2).

**Table 2:** Association between lung damage, ARDS, and mortality at 30 days.

Tomographic lung damage in acute respiratory distress syndrome					
ARDS Classification	Pulmonary damage severity index	Number of patients	Deaths	Censored	
				N	Percent
No ARDS	mild	96	1	95	99.0%
	moderate	128	14	114	89.1%
	severe	27	20	7	25.9%
	Total	251	35	216	86.1%
Mild ARDS	mild	22	1	21	95.5%
	moderate	69	20	49	71.0%
	severe	27	25	2	7.4%
	Total	118	46	72	61.0%
Moderate ARDS	mild	6	1	5	83.3%
	moderate	41	28	13	31.7%
	severe	39	39	0	0.0%
	Total	86	68	18	20.9%
Severe ARDS	moderate	17	16	1	5.9%
	severe	18	18	0	0.0%
	Total	35	34	1	2.9%
Overall		490	183	307	62.7%

ARDS: acute respiratory distress syndrome. N: number.

**Table 3:** Mortality at 30 days in patients with ARDS according to tomographic lung damage.

Survival pairwise comparisons Breslow (Generalized Wilcoxon)							
ARDS Classification	Pulmonary damage severity index	mild		moderate		severe	
		Chi-Square	Sig.	Chi-Square	Sig.	Chi-Square	Sig.
No ARDS	mild			1.690	.194	1.628	.202
	moderate	1.690	.194			.008	.930
	severe	1.628	.202	.008	.930		
Mild ARDS	mild			2.030	.154	1.418	.234
	moderate	2.030	.154			.459	.498
	severe	1.418	.234	.459	.498		
Moderate ARDS	mild			2.439	.118	**7.000**	**.008**
	moderate	2.439	.118			.007	.934
	severe	7.000	.008	.007	.934		
Severe ARDS	moderate					3.490	.062
	severe			3.490	.062		

ARDS: acute respiratory distress syndrome.

## 8 Discussion

This is the first report to associate acute respiratory distress syndrome, lung damage, quantified via a CT severity index, and mortality at 30 days. Previous reports have studied a wide range of factors that contribute to a poor outcome in COVID-19, such as advanced age, cardiovascular disease, chronic obstructive pulmonary disease, diabetes, and obesity [15]. We report a consecutive case series of 490 patients diagnosed with COVID-19 infection and with computed tomography, as well as several disease-severity scales to assess the need for mechanical ventilation, and mortality at 30 days. Our results suggest, that APACHE IV (HR, 1.191, 95% CI [1.126, 1.260]), SOFA (HR, 5.178, 95%CI [3.103, 8.641]), and CCI (HR, 0.673, 95%CI [0.510, 0.889]), as well as the pulmonary damage severity index (HR, 1.282, 95%CI [1.151, 1.428]), predict the need for invasive mechanical ventilation. However, the survival distribution between patients who required invasive mechanical ventilation and those who did not, between severe and critical pneumonia, between different levels of acute respiratory distress syndrome were not statistically different. Only moderate ARDS patients with mild and severe lung disease showed different 30-day mortality distributions. Although the survival distributions did not vary significantly, an overwhelming majority of patients (i.e., 84.35%) with a higher pulmonary damage severity index (i.e., 23>) died within 30 days of hospital admission, while only 25.91% with moderate lung damage and 2.42% with mild lung damage.

Previous studies have reported, up to 56% of normal lung CT scans in the early in COVID-19 (i.e., 0–2 days) and the findings most frequently observed were consolidation, bilateral involvement (i.e., 28% in the early stage, 76% in the intermediate stage, and 88% in the late stage) and peripheral, “Crazy-paving” ground-glass and reverse halo sign [16]. A study comparing tomographic findings associated to viral pneumonias (i.e., COVID-19 versus non-COVID-19, n = 424) reported greater frequency of peripheral lesions (i.e., 80% vs. 57%, p < 0.001), ground-glass opacities (i.e., 91% vs. 68%, p < 0.001), presence of fine reticular opacities (i.e., 56% vs. 22%, p < 0.001), vascular thickening pattern (i.e., 59% vs. 22%, p < 0.001) in patients with SARS-CoV-2 than in non-COVID-19 infection; conversely, central and peripheral distribution (i.e., 14% vs. 35%, p < 0.001), pleural effusion (i.e., 4.1% vs. 39%, p < 0.001), and lymphadenopathies (i.e., 2.7% vs. 10.2%, p < 0.001) were more frequent in non-COVID-19 than in COVID-19 infection [10]. Here, we report 89.80% of patients had ground-glass opacities, 81.63% had a radiologic consolidation sign, 42.45% had a vascular thickening pattern, 37.55% had lymphadenopathies, 14.90% had pleural effusion, and 2.65% had pulmonary thrombosis. Meanwhile, bilateral lesions were more common 91.02%, 85.51% had peripheral lesions and 75.92% had basal lobe lesions. Another study comparing lung CT findings between COVID-19 and influenza (n = 122), reported interlobular septal thickening in 66% of the patients, lineal opacities in 64%, ground-glass opacities and consolidation sign in 51%, bronchiolar wall thickening in 49%, only ground-glass opacities in 36%, and round opacities in 35% [17]. Barbosa and colleagues (2020) reported that pulmonary opacities with an extension greater than 50% and the presence of interlobular septal thickening were more frequent in patients admitted to the intensive care unit [18]. Furthermore, interlobular septal thickening, peripheral lesion distribution, pleural effusion, and lesion with an extension greater than 25% were associated with blood oxygen saturation of ≤93% [18]. Disease severity has also been liked to certain radiological signs and patterns. Consolidation radiological signs, linear opacities, ground-glass opacities, and bronchial thickening is observed more often in COVID-19 patients with severe or critical pneumonia patients, as well as a higher pulmonary damage severity index score (p < 0.001) than in patients without severe or critical pneumonia [19].

The main factor associated with disease severity and worst outcomes among COVID-19 patients was respiratory compromise upon hospital admission [5]. A comparative analysis between chest CT and chest x-rays in 1014 patients with COVID-19 reported a sensitivity, specificity, and diagnostic accuracy of 97%, 25%, and 68%, respectively, and a positive predictive value of 65% and a negative predicted value of 83% [20]. Greater lung damage (i.e., a score of 15 or greater assessed with the pulmonary damage severity index) has been associated with a poor clinical outcome at five days than patients with lower pulmonary damage severity index (i.e., a score = 8, range 8–11, p < 0.001), with a poor prognosis at five days in patients with a severity score ≥13 (i.e., sensitivity 80%, specificity 85.2%, AUC 0.853, HR 44.243, p < 0.001, 95CI [8.61, 227.37] [14]. In this study, we report two outcomes, the need for invasive mechanical ventilation and mortality at 30 days. We observed that as the pulmonary damage severity index increased the risk of mechanical ventilation did as well. Furthermore, while 84.25% of patients with severe lung damage, 25.91% of patients with moderate lung damage, and 2.42% of patients with mild lung damage died within 30 days, only in patients with moderate ARDS the survival distributions showed significant differences between mild and severe lung damage. A previous study (n = 201) of COVID-19 patients that developed ARDS, reported that older age (hazard ratio, HR, 3.26; 95% CI [2.08, 5.11]), neutrophilia (HR, 1.14; 95% CI [1.09, 1.19]), organ and coagulation dysfunction (e.g., higher lactate dehydrogenase, HR, 1.61; 95% CI [1.44, 1.79]) and D-dimer (HR, 1.03; 95% CI [1.01,1.04]) were associated with development of ARDS [21]. The overall mortality in COVID-19 critically ill patients ranges between 16 and 78% [22]. Mortality at 14 days in critically ill patients has been reported at 50% [23], while in the critical care unit patient's mortality was reported as 31% with a mean stay of 12 days (range 6–21) [24]. Among the factors associated with severe and critical pneumonia are age, diabetes, and chronic obstructive pulmonary disease [19]. All four severity scales (i.e., APACHE IV, SOFA, CCI, and pulmonary damage severity index) were different between patients who had severe and critical pneumonia upon admission to the critical care department. However, no statistical differences were observed in the survival distributions of patients with severe and critical pneumonia.

## 9 Limitations

One of the major limitations of this study is the lack of radiological follow-up, which limits the interpretability of the results as we can only make inferences about the initial radiological evaluation and not about disease progression. Another major limitation of this study was that some of the patients included received prior treatment with antibiotics and corticosteroids that could modify the tomographic findings. Associated with this limitation, we did not assess the role of coinfections in our sample, as a coinfection could confound the CT results. Furthermore, no lung biopsies were performed to confirm tomographic findings. A limitation associated with the analysis was the skewness of the pulmonary damage severity index scores, as a vast majority of the participants included in this study had a high severity index score, thus limiting the interpretability of the results presented here. Furthermore, as no statistically significant differences were observed in the survival distributions a Cox regression was not performed to determine the factor associated with the increased mortality.

## 10 Conclusion

All four disease severity scales (i.e., APACHE IV, SOFA, CCI, and pulmonary damage severity index) were associated with the need for invasive mechanical ventilation. We also report a frequency of 89.80% for ground-glass opacities, 81.63% for radiologic consolidation sign, 42.45% for vascular thickening pattern, 37.55% for lymphadenopathies, 14.90% for pleural effusion, and 2.65% for pulmonary thrombosis. We observed that 84.25% of patients with severe lung damage, 25.91% of patients with moderate lung damage, and 2.42% of patients with mild lung damage died within 30 days, and only in patients with moderate ARDS had different survival distributions between mild and severe lung damage. The survival distributions were similar between patients who needed invasive mechanical ventilation versus those who did not, between severe and critical pneumonia, and between different levels of ARDS.

## Declaration of competing interest

None of the authors report conflicts of interest. This research did not receive any specific grant from funding agencies in the commercial sector.

## References

[1] World Health Organization Director-General's remarks at the media briefing on 2019-nCoV on 11 February 2020. Director general's remarks at the media briefing on 2019-nCoV on 11 February 2020. https://www.who.int/es/emergencies/diseases/novel-coronavirus-2019.

[2] Centers for Disease Control and Prevention (2019). Novel coronavirus, wuhan, China. Information for healthcare professionals. https://www.cdc.gov/coronavirus/2019-nCoV/hcp/index.html.

[3] Tang J.W., Tambyah P.A., Hui D.S.C. (2020). Emergence of a novel coronavirus causing respiratory illness from Wuhan, China. J. Infect..

[4] Habibzadeh P., Stoneman E.K. (2020). The novel coronavirus: a bird's eye view. Int. J. Occup. Environ. Med..

[5] Guan W.J., Ni Z.Y., Hu Y. (2020). Clinical characteristics of coronavirus disease 2019 in China. N. Engl. J. Med..

[6] Wang D., Hu B., Hu C. (2020). Clinical characteristics of 138 hospitalized patients with 2019 novel coronavirus-infected pneumonia in wuhan, China [published correction appears in JAMA. 2021 mar 16;325(11):1113]. JAMA.

[7] Hanson K.E., Caliendo A.M., Arias C.A. (2020). Infectious diseases society of America guidelines on the diagnosis of COVID-19 [published online ahead of print, 2020 jun 16]. Clin. Infect. Dis..

[8] Upchurch C.P., Grijalva C.G., Wunderink R.G. (2018). Community-acquired pneumonia visualized on CT scans but not chest radiographs: pathogens, severity, and clinical outcomes. Chest.

[9] Zu Z.Y., Jiang M.D., Xu P.P. (2020). Coronavirus disease 2019 (COVID-19): a perspective from China. Radiology.

[10] Bai H.X., Hsieh B., Xiong Z. (2020). Performance of radiologists in differentiating COVID-19 from non-COVID-19 viral pneumonia at chest CT. Radiology.

[11] Liu C., Ye L., Xia R. (2020). Chest computed tomography and clinical follow-up of discharged patients with COVID-19 in wenzhou city, Zhejiang, China. Ann Am Thorac Soc.

[12] Graney B.A., Fischer A. (2019). Interstitial pneumonia with autoimmune features. Ann Am Thorac Soc.

[13] Chang Y.C., Yu C.J., Chang S.C. (2005). Pulmonary sequelae in convalescent patients after severe acute respiratory syndrome: evaluation with thin-section CT. Radiology.

[14] Mahdjoub E., Mohammad W., Lefevre T., Debray M.P., Khalil A., Study Group§ (2020). Admission chest CT score predicts 5-day outcome in patients with COVID-19. Intensive Care Med..

[15] Gandhi R.T., Lynch J.B., Del Rio C. (2020). Mild or moderate covid-19. N. Engl. J. Med..

[16] Bernheim A., Mei X., Huang M. (2020). Chest CT findings in coronavirus disease-19 (COVID-19): relationship to duration of infection. Radiology.

[17] Liu M., Zeng W., Wen Y., Zheng Y., Lv F., Xiao K. (2020). COVID-19 pneumonia: CT findings of 122 patients and differentiation from influenza pneumonia. Eur. Radiol..

[18] Barbosa C.S., Chaves G.W.O.G., de Oliveira C.V. (2020). COVID-19 pneumonia in the emergency department: correlation of initial chest CT findings with short-term outcome. Emerg. Radiol..

[19] Li K., Wu J., Wu F. (2020). The clinical and chest CT features associated with severe and critical COVID-19 pneumonia. Invest. Radiol..

[20] Ai T., Yang Z., Hou H. (2020). Correlation of chest CT and RT-PCR testing for coronavirus disease 2019 (COVID-19) in China: a report of 1014 cases. Radiology.

[21] Wu C., Chen X., Cai Y. (2020). Risk factors associated with acute respiratory distress syndrome and death in patients with coronavirus disease 2019 pneumonia in wuhan, China [published correction appears in JAMA intern med. 2020 jul 1;180(7):1031]. JAMA Intern. Med..

[22] Grasselli G., Greco M., Zanella A. (2020). Risk factors associated with mortality among patients with COVID-19 in intensive care units in Lombardy, Italy [published correction appears in JAMA intern med. 2021 jul 1;181(7):1021]. JAMA Intern. Med..

[23] Bhatraju P.K., Ghassemieh B.J., Nichols M. (2020). Covid-19 in critically ill patients in the seattle region - case series. N. Engl. J. Med..

[24] Ferrando C., Mellado-Artigas R., Gea A. (2020). Patient characteristics, clinical course and factors associated to ICU mortality in critically ill patients infected with SARS-CoV-2 in Spain: a prospective, cohort, multicentre study. Características, evolución clínica y factores asociados a la mortalidad en UCI de los pacientes críticos infectados por SARS-CoV-2 en España: estudio prospectivo, de cohorte y multicéntrico. Rev. Esp. Anestesiol. Reanim..

