# Supplementary material for: Tomographic findings and mortality in patients with severe and critical pneumonia with COVID-19 diagnosis
Source: Respir Med Case Rep. 2022 Oct 5;40:101752. doi: 10.1016/j.rmcr.2022.101752 (PMC9534537; doi:10.1016/j.rmcr.2022.101752)
Supplement: Multimedia component 1 [file mmc1.docx]

**Supplementary material**

**Supplementary Figure 1. Survival as a function of mechanical ventilation**


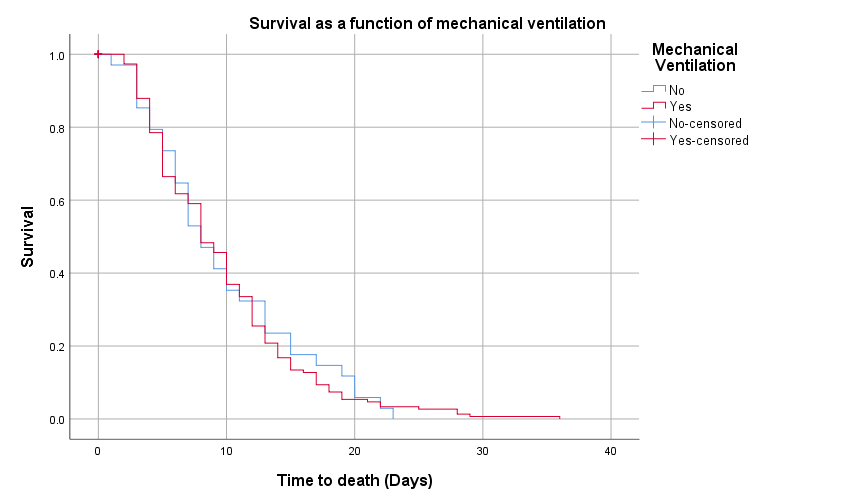


**Supplementary Figure 2. Survival as a function of pneumonia severity**

**
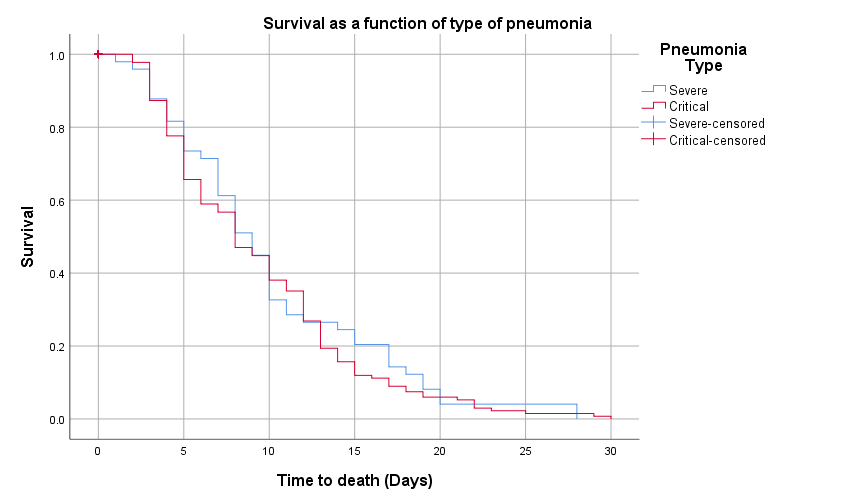
**

**Supplemental Figure 3. Survival as a function of type of pneumonia**


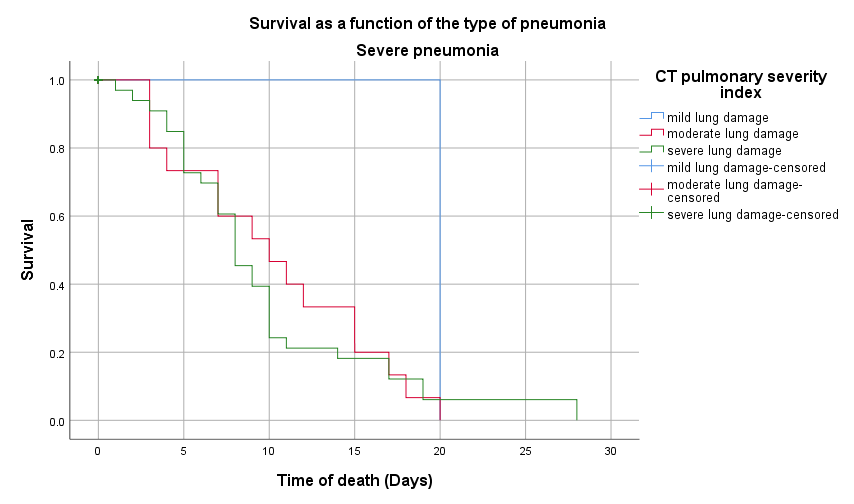
A)


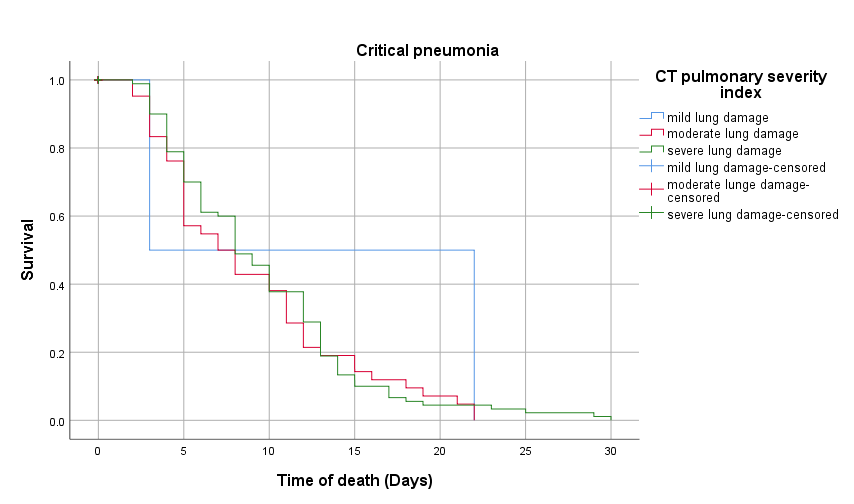


B)

**CT: computed tomography.**

**Supplemental Figure 4. Survival as a function of acute respiratory distress syndrome**


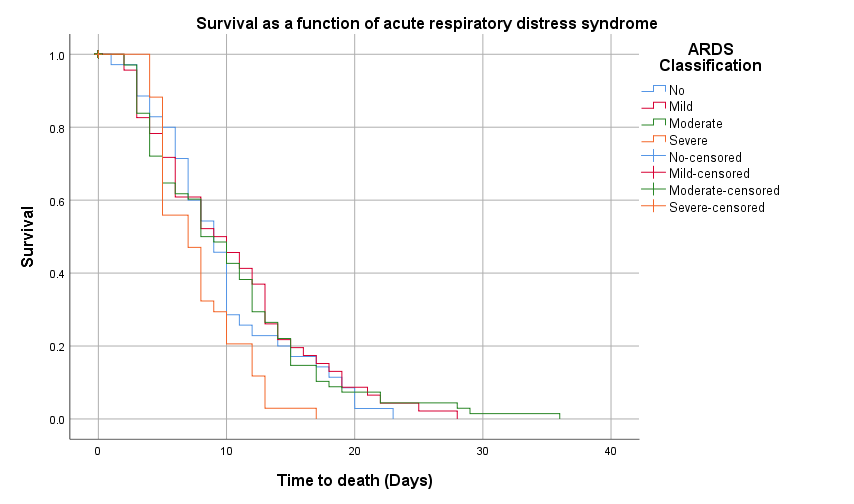


**ARDS: acute respiratory distress syndrome.**
